# Predictors of Engagement in Multiple Modalities of Digital Mental Health Treatments: Longitudinal Study

**DOI:** 10.2196/48696

**Published:** 2024-11-07

**Authors:** Molly Aideen Nowels, Meghan McDarby, Lilla Brody, Evan Kleiman, Sara Sagui Henson, Cynthia Castro Sweet, Elissa Kozlov

**Affiliations:** 1 Division of Geriatrics and Palliative Medicine Weill Cornell Medicine New York, NY United States; 2 Department of Psychiatry & Behavioral Sciences Memorial Sloan Kettering Cancer Center New York City, NY United States; 3 Divison of Geriatrics & Palliative Medicine Weill Cornell Medicine New York City, NY United States; 4 Department of Psychology Rutgers University New Brunswick, NJ United States; 5 Modern Health San Francisco, CA United States

**Keywords:** digital health, mental health, health care benefit, prediction, technology, digital mental health, employer-based, teletherapy, coaching, utilization, mobile phone

## Abstract

**Background:**

Technology-enhanced mental health platforms may serve as a pathway to accessible and scalable mental health care; specifically, those that leverage stepped care models have the potential to address many barriers to patient care, including low mental health literacy, mental health provider shortages, perceived acceptability of care, and equitable access to evidence-based treatment. Driving meaningful engagement in care through these platforms remains a challenge.

**Objective:**

This study aimed to examine predictors of engagement in self-directed digital mental health services offered as part of an employer-based mental health benefit that uses a technology-enabled care platform.

**Methods:**

Using a prospective, longitudinal design, we examined usage data from employees who had access to an employer-sponsored mental health care benefit. Participants had access to a digital library of mental health resources, which they could use at any time, including daily exercises, interactive programs, podcasts, and mindfulness exercises. Coaching and teletherapy were also available to. The outcome was engagement with the self-directed digital mental health resources, measured by the number of interactions. Poisson regression models included sociodemographic characteristics, patient activation, mental health literacy, well-being, PHQ-9 and GAD-7 scores at baseline, primary concern for engaging in treatment, and the use of coaching or teletherapy sessions.

**Results:**

In total 950 individuals enrolled in the study, with 38% using any self-directed digital mental health resources. Approximately 44% of the sample did not use the app during the study period. Those using both self-directed digital and 1:1 modalities made up about one-quarter of the sample (235/950, 24.7%). Those using only coaching or therapy (170/950, 17.9%) and those using only self-directed digital mental health resources (126/950, 13.3%) make up the rest. At baseline, these groups statistically significantly differed on age, PHQ-9, GAD-7, MHLS, and primary concern. Receipt of coaching and teletherapy was associated with the number of self-directed digital mental health resources interactions in adjusted Poisson regression modeling. Use of any coach visit was associated with 82% (rate ratio [RR] 1.82, 95% CI 1.63-2.03) more self-directed digital mental health resource interactions while use of any teletherapy session was associated with 80% (RR 1.80, 95% CI 1.55-2.07) more digital mental health resources interactions (both *P*<.001). Each additional year of age was associated with increased digital mental health resources interactions (RR 1.04, 95% CI (1.03-1.05), and women had 23% more self-directed digital resources interactions than men (RR 1.23, 95% CI 1.09-1.39).

**Conclusions:**

Our key finding was that the use of coaching or teletherapy was associated with increased self-directed digital mental health resource use. Higher self-directed digital resource engagement among those receiving coaching or therapy may be a result of provider encouragement. On the other hand, when a participant engages with 1 modality in the platform, they may be more likely to begin engaging with others, becoming “super users” of all resources.

## Introduction

Mental health and substance use disorders are leading causes of disease burden in the United States [1]. However, while nearly 25% of adults experience a mental disorder at any given time [2], less than a quarter (21.7%) of individuals with a mental disorder receives treatment from a licensed mental health clinician [3]. This discrepancy between the prevalence of mental health disorders and rates of mental health service use is largely attributed to barriers to health care access, such as not being able to schedule an appointment in a timely manner [4] and persistent shortages in the mental health workforce—especially in rural communities and for people with serious mental illness [5,6]. As the demand for psychological services outpaces the supply of mental health providers, innovative mental health care delivery models are increasingly needed.

Digital mental health (digital health) platforms represent a promising approach to deliver accessible, scalable, and timely mental health care. These platforms often provide care through multiple modalities with varying intensities (eg, traditional one-on-one telepsychotherapy or telecoaching, asynchronous self-guided treatments, and telegroup-based treatments). As digital health platforms can be equipped to provide a range of treatments to patients, they have the potential to dismantle key barriers to patient care, including mental health provider shortages and inequitable access to mental health treatment [7-9].

However, digital health platforms can be challenging to implement, largely because user adoption and continued engagement are relatively low [10-12]. While traditional psychotherapy also struggles with early dropout rates ranging between 8% and 20% [13,14], digital health historically has seen much higher rates of discontinuation [11]. As a result, while they hold the potential to promote increased, equitable access to mental health care, additional research is needed to determine how to facilitate increased engagement in mental health care from digital health platforms. Recent research investigating engagement in digital health applications after initial adoption suggests that several factors, including user attributes (eg severity of depressive symptoms), technology issues (eg concerns about anonymity and privacy), components of user experience (eg, interface design of a digital health application) [15] and motivation to engage in the health goal of interest, are associated with continued use [16]. Other research indicates that users’ beliefs about the severity of their health conditions may also be associated with digital health adoption and continued use [17] and that improvement in psychological outcomes of interest during early treatment may predict continued engagement [18]. Finally, it is important to understand the appropriate level of engagement in different services given a user’s needs and preferences. Not all users will need long-term unlimited engagement with a digital health platform, and engagement likely involves evaluating the need for continued use [19,20].

As the number of digital mental health platforms continues to grow and more modalities of digital mental health care (eg, podcasts, online courses, meditations, discussion boards, etc.) become available to consumers, it is critical to understand predictors of engagement in digital mental health care. Elucidating the range of factors that predict initial and continued use of digital health services for mental health will support these platforms in further customizing care approaches to maximize user engagement, and ultimately, optimize personalized mental health treatment. The purpose of this study was to identify predictors of engagement in multiple modalities of mental health treatments offered on an employer-sponsored digital mental health care benefit.

## Methods

### Design and Participants

This investigation was part of a prospective, longitudinal, observational study of individuals who received services through an employer-sponsored digital mental health benefits platform (Modern Health Inc). The study timeframe was from September 20, 2021, to May 31, 2022.

Eligible participants were 18 years or older, based in the United States, registered for the benefit, and had access to a smartphone, tablet, or computer.

### Procedures

**Figure 1:**
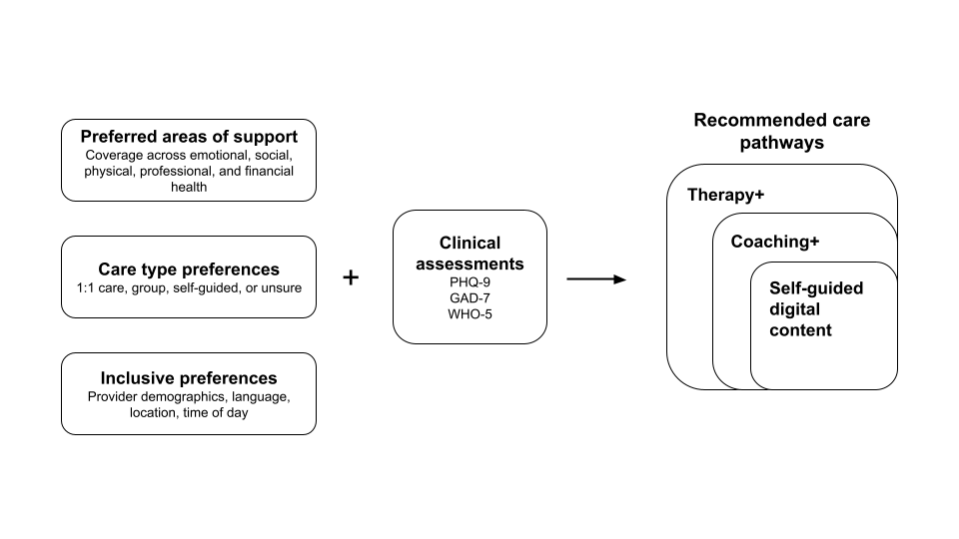
Stratified stepped care model incorporating areas of support, care preferences, and clinical assessments into personalized care recommendations. PHQ-9: 9-item Patient Health Questionnaire; GAD-7: 7-item Generalized Anxiety Disorder Questionnaire; WHO-5: 5-item World Health Organization Well-Being Index.

Eligible employees registered for a Modern Health account through a mobile app or website and completed several onboarding assessments, which asked about areas of focus, care modality preferences, and clinical symptoms. The platform’s proprietary algorithm recommended an initial care pathway to participants based on this information. Recommendations used a stratified stepped care model, meaning that patients with higher acuity and with certain areas of focus were more likely to be recommended to more intensive care options (eg, one-on-one teletherapy) (more details in Figure 1). Participants could use their recommended care or self-refer or be referred by a provider to any combination of care.

Members who matched with a provider or used at least 1 digital resource after onboarding were invited by email to complete a screening questionnaire within 2 weeks of onboarding, which contained questions about demographics. We used the demographic screening information for purposeful recruitment to ensure representation in racial and ethnic identities, gender identities, socioeconomic status, age groups, and mental health symptoms at baseline. After the screening questionnaire, eligible participants were directed to a consent form to provide informed consent. Consenting participants were sent the baseline survey asking questions about their mental, social, and physical health by email along with instructions and compensation. Participants were sent a follow-up survey at 12 weeks post baseline that contained the same mental, social, and physical health questions. All forms and surveys were hosted on Qualtrics’ online survey platform.

### Digital Mental Health Services

Individuals in this study were able to engage in all of the following digital mental health services, within the limits of their employer-covered sessions for 1:1 care modalities. Self-directed digital resources had no limits.

### Digital Resources

Participants had access to a digital library of programs and resources they could use in unlimited quantities at any time. These included daily meditative exercises, interactive programs and podcasts, mindfulness exercises like meditations and breathing exercises, and self-paced structured educational lessons (similar to self-help workbooks). All digital materials were developed and designed by an in-house team of clinical psychologists. Digital health programs were designed to cover topics such as emotions, relationships, professional life, healthy lifestyles, and finances. Engagement across all digital resources was combined in analyses to represent total digital program engagement.

### Telecoaching

Coaches were certified by an International Coaching Federation accredited program and underwent a careful screening process by Modern Health. They provided 30-minute telecoaching services to eligible employees through a videoconferencing platform (Zoom; Zoom Video Communications). Coaches and participants were also able to communicate by messaging within the app. All coaches had at least 150 hours of coaching experience in addition to completing additional training in evidence-based techniques and Modern Health’s proprietary model of care. Rather than being confined to any specific protocol, coaches were encouraged to implement evidence-based techniques where appropriate. The number of coaching sessions attended by participants depended on the number of sessions covered by their employer, as well as on personal preferences and their level of need.

### Teletherapy

Furthermore, 50 minutes one-on-one teletherapy sessions were provided through a videoconferencing platform (Zoom) by licensed and vetted therapists who had an advanced degree in clinical psychology or a related field (eg, PhD, PsyD, LCSW, LMFT, or LPC). Similar to telecoaching, therapists and participants were able to communicate through messaging in-app. All therapists were skilled in evidence-based practices, such as cognitive behavioral therapy, acceptance and commitment therapy, and dialectical behavior therapy and had completed additional training in Modern Health’s proprietary model of care. Similar to telecoaching, the number of therapy sessions attended by participants depended on the number of sessions covered by their employer, as well as on personal preferences and their level of need [21].

### Outcome Measures

Engagement in digital mental health services: We created 4 outcome variables to capture engagement in platform services. First, we created a 4-level categorical variable to represent use of the digital health services: (1) only self-directed digital resource use, (2) only telecoach or teletherapist use, (3) use of both digital and telecoach or teletherapist modalities, and (4) no use of any modality. Second, we created a binary variable (0/1) indicating whether participants used at least 1 service within the platform (inclusive of self-directed digital resources, one-on-one telecoaching visits, and one-on-one teletherapy visits). Third, we created a binary variable (0/1) indicating whether participants used at least one of the self-directed digital resources collapsed across resource types: meditative exercises, programs, meditations, and podcasts. Fourth, we created a count of the number of self-directed digital resources each participant used. The use of both self-directed digital resources and 1:1 modalities was collected automatically by the digital mental health platform. Engagement data during the study period (between the baseline and follow-up surveys) was extracted from the platform for analysis.

### Predictor Measures

#### Demographics

Participants self-identified their age, race and ethnicity (American Indian or Alaskan Native, Asian, Black or African American, Hawaiian or Pacific Islander, Hispanic, Latino, or Spanish origin, non-Hispanic white, prefer to self-describe, multiracial; collapsed to non-Hispanic White vs non-White), gender identity (man, woman, non-binary), and educational attainment (less than a bachelors’ degree, bachelors’ degree, and more than a bachelors’ degree).

#### Depression

The 9-item Patient Health Questionnaire (PHQ-9) [22] was used to assess the presence of depression symptoms over the past 2 weeks. Participants responded on a 4-point scale (0= “Not at all,” 3= “Nearly every day”), and total scores were categorized into 1 of 5 severity ratings: none, minimal, mild, moderate, or moderately severe. Higher scores indicate higher severity of depression symptomatology.

#### Anxiety Symptoms

The 7-item Generalized Anxiety Disorder Questionnaire (GAD-7) [23] was used to assess the presence of anxiety symptoms over the past 2 weeks. Participants responded on a 4-point scale (0=“Not at all,” 3=“Nearly every day”) and total scores were categorized into 1 of 4 severity ratings: minimal, mild, moderate, or severe. Higher scores indicate higher severity of anxiety symptomatology.

#### Well-Being

The 5-item World Health Organization Well-Being Index (WHO-5) [24] was used to assess well-being over the past 2 weeks. This construct was assessed during the onboarding process, approximately 2 weeks before the baseline assessment. Participants responded on a 6-point scale (0=“At no time,” 5=“All of the time”). Higher scores indicate greater subjective well-being.

#### Patient Activation

The 13-item Patient Activation Measure for Mental Health (PAM-MH) was used [25]. Participants chose from 5 possible response options ranging from 1=Strongly Disagree to 4=Strongly Agree to 5=Not Applicable. Higher scores indicate higher patient activation.

#### Mental Health Literacy

Mental health literacy was measured using 13 items from the Mental Health Literacy Scale (MHLS) [26]. Response options ranged from 1=Strongly Disagree to 5=Strongly Agree.

#### Area of Focus

Participants were able to choose from over 40 topics of focus, or reasons for registering for the platform, during the onboarding process. These topics were wide-ranging and organized by the following well-being dimensions: “my emotions,” “my professional life,” “my physical well-being,” “my relationships,” and “my finances.”

#### One-on-One Care Engagement

Variables indicating whether participants had used at least telecoaching (0/1) or teletherapy (0/1) sessions within the platform were also included as predictors in analyses predicting the use of self-directed digital resources.

### Statistical Analyses

We conducted 4 sets of analyses examining the association between all the predictors and each engagement in digital mental health services outcome. First, we used descriptive analyses to investigate differences in predictor variables across the 4-level categorical variable of use, with ANOVA performed for continuous predictors and chi-square tests for categorical predictors.

Second, we examined predictors of any digital health service use in the app using logistic regression. The outcome variable was the binary variable representing if participants had used at least 1 service. In this regression model, we included age, race and ethnicity (non-White as reference group), gender identity (man as reference group), educational attainment (less than bachelor’s degree completion as reference group), patient activation, mental health literacy, well-being, area of focus (“my emotions” as reference group), baseline depression (entered as a linear predictor), and baseline anxiety (entered as a linear predictor) as predictors.

Third, we examined predictors of any digital health self-directed digital resource use in the app using logistic regression. The outcome variable was the binary variable representing if participants had at least one self-directed digital resource in the 3-month follow-up period. In this regression, we included age, race and ethnicity, gender identity, educational attainment, patient activation, mental health literacy, well-being, primary concern, baseline depression (entered as a linear predictor), and baseline anxiety (entered as a linear predictor) as predictors, with the same reference groups as in the previous analysis.

Fourth, using Poisson regression, we examined if the use of any telecoaching or teletherapy visits was associated with the number of self-directed digital resources used. We created binary variables for both telecoaching and teletherapy, reflecting any use of each modality in the follow-up period. We used both unadjusted and adjusted approaches to investigate this association. In the unadjusted model, we only included the telecoaching and teletherapy visit variables as predictors. In the adjusted model, we additionally included age, race and ethnicity, gender identity, educational attainment, patient activation, mental health literacy, well-being, and area of focus, with reference groups mirroring the previous analyses. We also performed the same adjusted Poisson regression analyses on a subgroup of our sample: those with elevated GAD-7 or PHQ-9 scores (scores on either scale above 9 indicating moderate symptoms [27,28]) at baseline.

### Ethical Considerations

All participants provided informed consent, and this study protocol was reviewed and approved by the Western Clinical Group institutional review board (protocol #1316167). Compensation for this study was a US $25 digital gift card per completed survey. All data were deidentified.

## Results

In total, 950 individuals were enrolled in this study. More than half of participants (n=531, 55.9%) engaged with at least 1 service within the platform (eg, self-directed digital resource, telecoach, or teletherapist session); 44.1% (n=419) did not engage in any service. Among engagers, 44.3% (n=235) used both self-directed digital resources and 1:1 care (teletherapy or telecoaching), 32% (n=170) used only 1:1 care, and 23.7% (n=126) used only self-directed digital resources. At baseline, these engagement groups statistically significantly differed in age, depressive symptoms, anxiety symptoms, health literacy, and area of focus (Table 1). Among participants who only used self-directed digital resources, the average number of digital engagements was 2.0 (median 1.0). Among those using both 1:1 care and self-directed digital resources, the average number of digital engagements was 3.4 (median 2.0).

In the logistic regression analysis predicting engagement in any service, only higher educational attainment was associated with increased odds of engaging in at least 1 service (Table 2). Relative to participants without a bachelor’s degree, those with a bachelor’s had 54.5% (*P=.*03) higher odds of engaging, and those with education greater than a bachelor’s had 56.2% (*P=.*05) higher odds of engaging. No other variables in the model were statistically significant.

In our logistic regression analysis predicting engagement in any self-directed digital resources, certain demographic characteristics were associated with an increased likelihood of engagement. For every year of increasing age, subjects were 2.6% more likely to have used any self-directed resources (*P=.*003). Relative to men, women had 45.4% higher odds of using any self-directed resources (*P=.*01). Finally, educational attainment was also significantly associated with self-directed resource use, such that those with bachelor’s degrees (odds ratio 1.56 95% CI 1.026-2.414; *P=.*04) and with education higher than a bachelor’s degree (odds ratio 1.65 95% CI 1.045-2.625; *P=.*03) had higher odds of self-directed resource engagement than those with less than a bachelor’s degree (Table 3).

In an unadjusted Poisson regression examining if engagement in at least 1 telecoaching or teletherapy was associated with self-directed digital resources use, engagement in any telecoach sessions was associated with a 71.6% increase in the number of self-directed digital resources used, while engagement in any teletherapy sessions was associated with a 41.4% increase in the number of digital resources used (Table 4). After adjusting for the additional predictor variables described above, engagement in any telecoaching sessions was associated with an 82% increase in the number of self-directed resources used, and engagement in any teletherapy sessions was associated with a 79.5% increase in the number of digital resources used. In the adjusted analysis, age was a significant predictor of engagement, with each additional year of age associated with a 3.9% increase in the number of self-directed digital resources used. Relative to men, those identifying as women used 23% more digital resources and non-binary individuals did not statistically significantly differ. The primary topic of focus was also a significant predictor of self-directed resource engagement, with finances (relative risk [RR] 1.46, *P=*.02), physical well-being (RR 2.28 *P*<.001), professional life (RR 1.71, *P*<.001), and relationships (RR 1.37, *P*<.001) all being associated with increased digital resource use as compared with those who chose emotions. In the subgroup analysis conducted in participants with an elevated GAD-7 or PHQ-9 score, engagement in any telecoaching was associated with a 101% increase in the number of self-directed digital resources used (*P*<*.*001), and engagement in any teletherapist visit was associated with a 77.8% increase in the number of digital resources used (*P*<*.*001) (Table 5).

**Table 1:** Demographics and baseline characteristics, overall and by modality of app use. *P* values are derived from chi-square tests for categorical variables and from ANOVA tests for continuous variables.

			Overall (N=950)		No use (N=419)		Therapy or coach only (N=170)		Self-directed digital resources only (N=126)		Both modalities (N=235)		*P* value	
**Age**														<*.*001
	Mean (SD)	33.9 (8.70)		33.9 (8.68)		31.5 (7.02)		36.8 (9.79)		34.0 (8.76)				
	Median (range)	32 (19-65)		32 (19-63)		30 (22-57)		36.5 (22-60)		32 (21-65)				
**Race and ethnicity (White vs non-White), n (%)**														*.*74
	Non-White	399 (42)		184 (43.9)		73 (42.9)		49 (38.9)		93 (39.6)				
	White	548 (57.7)		232 (55.4)		97 (57.1)		77 (61.1)		142 (60.4)				
	Missing	3 (0.3)		3 (0.7)		0 (0)		0 (0)		0 (0)				
**Gender identity, n (%)**														*.*66
	Gender-nonbinary	46 (4.8)		23 (5.5)		8 (4.7)		4 (3.2)		11 (4.7)				
	Man	343 (36.1)		164 (39.1)		59 (34.7)		48 (38.1)		72 (30.6)				
	Woman	559 (58.8)		230 (54.9)		103 (60.6)		74 (58.7)		152 (64.7)				
	Prefer not to say	2 (0.2)		2 (0.5)		0 (0)		0 (0)		0 (0)				
**Educational attainment, n (%)**														*.*40
	<Bachelors	140 (14.7)		75 (17.9)		23 (13.5)		17 (13.5)		25 (10.6)				
	Bachelors	545 (57.4)		230 (54.9)		105 (61.8)		71 (56.3)		139 (59.1)				
	>Bachelors	265 (27.9)		114 (27.2)		42 (24.7)		38 (30.2)		71 (30.2)				
**Baseline PHQ-9^a^**														.03
	Mean (SD)	8.58 (5.96)		9.13 (6.51)		8.51 (5.41)		7.11 (5.52)		8.54 (5.51)				
	Median (range)	8 (0-27)		8 (0-27)		8 (0-23)		6 (0-23)		8 (0-25)				
	Missing, n (%)	35 (3.7)		35 (8.4)		0 (0)		0 (0)		0 (0)				
**Baseline GAD-7^b^**														*.*01
	Mean (SD)	7.73 (5.51)		8.03 (5.75)		7.86 (5.21)		6.10 (5.09)		8.01 (5.44)				
	Median (range)	7 (0-21)		7 (0-21)		7 (0-21)		5 (0-20)		7 (0-21)				
	Missing, n (%)	34 (3.6)		34 (8.1)		0 (0)		0 (0)		0 (0)				
**Baseline PAM^c^**														*.*85
	Mean (SD)	58.3 (13.0)		57.9 (13.1)		59.3 (13.2)		58.3 (12.8)		58.2 (12.7)				
	Median (range)	55.6 (17.9-100)		53.2 (17.9-100)		55.6 (34.2-100)		55.6 (30.4-100)		55.6 (31.7-100)				
	Missing, n (%)	35 (3.7)		34 (8.1)		1 (0.6)		0 (0)		0 (0)				
**Baseline MHLS^d^**														.02
	Mean (SD)	55.9 (6.43)		55.2 (7.13)		56.9 (6.01)		56.9 (6.16)		55.8 (5.46)				
	Median (range)	57 (29-65)		57 (29-65)		58.5 (34-65)		58 (29-65)		57 (37-65)				
	Missing, n (%)	26 (2.7)		26 (6.2)		0 (0)		0 (0)		0 (0)				
**Baseline WHO-5^e^**														.16
	Mean (SD)	43.3 (18.3)		42.1 (17.7)		42.8 (19.4)		46.7 (18.7)		44.1 (18.2)				
	Median (range)	44.0 (0-100)		40 (0-100)		40 (4-88)		48 (8-84)		44 (0-100)				
**Any past mental health care, n (%)**														.88
	No	233 (24.5)		112 (26.7)		43 (25.3)		33 (26.2)		45 (19.1)				
	Yes	244 (25.7)		111 (26.5)		42 (24.7)		34 (27)		57 (24.3)				
	Missing	473 (49.8)		196 (46.8)		85 (50)		59 (46.8)		133 (56.6)				
**Any current mental health care, n (%)**														.66
	No	464 (48.8)		217 (51.8)		82 (48.2)		62 (49.2)		103 (43.8)				
	Yes	275 (28.9)		114 (27.2)		49 (28.8)		44 (34.9)		68 (28.9)				
	Missing	211 (22.2)		88 (21)		39 (22.9)		20 (15.9)		64 (27.2)				
**Primary concern, n (%)**														.02
	My emotions	466 (49.1)		198 (47.3)		102 (60)		44 (34.9)		122 (51.9)				
	My finances	33 (3.5)		15 (3.6)		5 (2.9)		5 (4)		8 (3.4)				
	My physical well-being	95 (10)		40 (9.5)		9 (5.3)		23 (18.3)		23 (9.8)				
	My professional life	141 (14.8)		65 (15.5)		17 (10)		27 (21.4)		32 (13.6)				
	My relationship	214 (22.5)		100 (23.9)		37 (21.8)		27 (21.4)		50 (21.3)				
	Missing	1 (0.1)		1 (0.2)		0 (0)		0 (0)		0 (0)				

^a^PHQ-9: 9-item Patient Health Questionnaire.

^b^GAD-7: 7-item Generalized Anxiety Disorder Questionnaire.

**^c^**PAM: Patient Activation Measure.

^d^MHLS: Mental Health Literacy Scale.

**^e^**WHO-5: 5-item World Health Organization Well-Being Index.

**Table 2:** Adjusted logistic regression analysis predicting any app use (N=908).

Variables		Odds ratio (95% CI)		*P* value
Age		1.002 (0.986-1.019)		.78
**Race and ethnicity**				
	White	1.074 (0.813-1.419)	.61	
	Non-White	REF^a^	—^b^	
**Gender identity**				
	Nonbinary	1.084 (0.559-2.125)	.81	
	Woman	1.310 (0.977-1.756)	.07	
	Man	REF	—	
**Educational attainment**				
	Greater than bachelors	1.562 (1.01-2.422)	.05	
	Bachelors	1.545 (1.037-2.305)	.03	
	Less than bachelors	REF	—	
**Patient activation**				
	PAM^c,d^	0.999 (0.987-1.01)	.82	
**Mental health literacy**				
	MHLS^c,e^	1.022 (0.999-1.045)	.07	
**Well-being**				
	WHO-5^c,f^	1.003 (0.994-1.013)	.51	
**Primary concern**				
	My emotions	REF	—	
	My finances	0.864 (0.409-1.871)	.70	
	My physical well-being	0.926 (0.577-1.495)	.75	
	My professional life	0.779 (0.52-1.171)	.23	
	My relationships	0.808 (0.568-1.151)	.24	
**Mental health at baseline**				
	PHQ-9^c,g^	0.975 (0.938-1.013)	.19	
	GAD-7^c,h^	1.005 (0.968-1.044)	.79	

^a^REF: reference range.

^b^Not applicable.

^c^PAM, MHLS, WHO-5, PHQ-9, and GAD-7 scores were entered as linear predictors.

^d^PAM: Patient Activation Measure.

^e^MHLS: Mental Health Literacy Scale.

^f^WHO-5: 5-item World Health Organization Well-Being Index.

^g^PHQ-9: 9-item Patient Health Questionnaire.

^h^GAD-7: 7-item Generalized Anxiety Disorder Questionnaire.

**Table 3:** Adjusted logistic regression analysis predicting any self-directed digital resources use (N=908).

Variable		Odds ratio (95% CI)	*P* value
Age		1.026 (1.009-1.044)	.003
**Race and ethnicity**			
	White	1.115 (0.841-1.48)	.45
	Non-White	REF^a^	—^b^
**Gender identity**			
	Non-binary	1.122 (0.55-2.22)	.75
	Woman	1.454 (1.079-1.965)	.01
	Man	REF	—
**Educational attainment**			
	Greater than bachelors	1.648 (1.045-2.625)	.03
	Bachelors	1.564 (1.026-2.414)	.04
	Less than bachelors	REF	—
**Patient activation**			
	PAM^c,d^	0.995 (0.983-1.007)	.40
**Mental health literacy**			
	MHLS^c,e^	1.003 (0.98-1.027)	.82
**Well-being**			
	WHO-5^c,f^	1.008 (0.998-1.017)	.11
**Primary concern**			
	My emotions	REF	—
	My finances	1.143 (0.524-2.43)	.73
	My physical well-being	1.432 (0.894-2.292)	.13
	My professional life	1.158 (0.769-1.737)	.48
	My relationships	0.989 (0.689-1.414)	.95
**Mental health at baseline**			
	PHQ-9^c,g^	0.992 (0.954-1.031)	.68
	GAD-7^c,h^	1.004 (0.967-1.043)	.83

^a^REF: reference.

^b^Not applicable.

^c^PAM, MHLS, WHO-5, PHQ-9, and GAD-7 scores were entered as linear predictors.

^d^PAM: Patient Activation Measure.

^e^MHLS: Mental Health Literacy Scale.

^f^WHO-5: 5-item World Health Organization Well-Being Index.

^g^PHQ-9: 9-item Patient Health Questionnaire.

^h^GAD-7: 7-item Generalized Anxiety Disorder Questionnaire.

**Table 4:** Unadjusted and adjusted Poisson regression models predicted the number of self-directed digital resources used.

Variable			Unadjusted model (N=950)				Adjusted model (N=911)		
			RR^a^ (95% CI)		*P* value		RR (95% CI)		*P* value
**Coaching or therapy**									
	Any coach sessions	1.716 (1.542-1.908)		<.001		1.820 (1.629-2.033)		<.001	
	Any therapy sessions	1.414 (1.247-1.6)		<.001		1.795 (1.554-2.068)		<.001	
Age			—^b^		—		1.039 (1.033-1.045)		<.001
**Race and ethnicity**									
	White	—		—		1.120 (0.998-1.258)		.06	
	Non-white	—		—		REF^c^		—	
**Gender identity**									
	Nonbinary	—		—		1.794 (1.425-2.236)		.95	
	Woman	—		—		1.230 (1.091-1.388)		<.001	
	Man	—		—		REF		—	
**Educational attainment**									
	Less than bachelors	—		—		REF		—	
	Greater than bachelors	—		—		0.991 (0.83-1.186)		.92	
	Bachelors	—		—		1.147 (0.975-1.356)		.10	
**Patient activation**									
	PAM^d,e^ score	—		—		0.991 (0.987-0.996)		<.001	
**Mental health literacy**									
	MHLS^d,f^ score	—		—		1.000 (0.991-1.01)		.97	
**Well-being**									
	WHO-5^d,g^ score	—		—		1.001 (0.998-1.004)		.52	
**Primary concern**									
	My emotions	—		—		REF		—	
	My finances	—		—		1.456 (1.058-1.954)		.02	
	My physical well-being	—		—		2.257 (1.925-2.64)		<.001	
	My professional life	—		—		1.713 (1.457-2.009)		<.001	
	My relationships	—		—		1.365 (1.174-1.584)		<.001	

^a^RR: rate ratio.

^b^Not available.

^c^REF: reference.

^d^PAM, MHLS, and WHO-5 scores were entered as linear predictors.

^e^PAM: Patient Activation Measure.

^f^MHLS: Mental Health Literacy Scale.

^g^WHO-5: 5-item World Health Organization Well-Being Index.

**Table 5:** Subgroup Poisson regression analysis conditional on elevated GAD-7 or PHQ-9 score (≥ 10) at baseline, predicting number of self-directed resources used (n=400).

Variable		RR^a^ (95% CI)	*P* value
**Use of therapy or coach**			
	Any coach visits	2.099 (1.76-2.503)	<.001
	Any therapist visits	1.778 (1.455-2.165)	<.001
Age		1.036 (1.025-1.047)	<.001
**Race and ethnicity**			
	Non-White	REF^b^	—^c^
	White	1.218 (1.014-1.467)	.04
**Gender identity**			
	Man	REF	—
	Woman	0.864 (0.71-1.053)	.14
	Non-binary	1.266 (0.859-1.819)	.22
**Educational attainment**			
	<Bachelors	REF	—
	>Bachelors	1.005 (0.758-1.339)	.97
	Bachelors	1.203 (0.943-1.553)	.15
**Patient activation**			
	PAM^d,e^	0.985 (0.977-0.993)	<.001
**Mental health literacy**			
	MHLS^d,f^	1.008 (0.993-1.023)	.31
**Well-being**			
	WHO-5^d,g^	0.993 (0.987-0.999)	.03
**Primary concern**			
	My emotions	REF	—
	My finances	2.015 (1.202-3.191)	.005
	My physical well-being	2.411 (1.865-3.095)	<.001
	My professional life	2.318 (1.814-2.945)	<.001
	My relationships	1.350 (1.04-1.735)	.02

^a^RR: rate ratio.

^b^REF: reference range.

^c^Not applicable.

^d^PAM, MHLS, and WHO-5 scores were entered as linear predictors.

^e^PAM: Patient Activation Measure.

^f^MHLS: Mental Health Literacy Scale.

^g^WHO-5: 5-item World Health Organization Well-Being Index.

## Discussion

### Principal Findings

Our study aimed to evaluate predictive factors of engagement in multiple digital health modalities offered by a digital mental health employer-based benefit. Over half of the participants (55.9%, 531/950) used at least 1 component of the digital mental health platform at least once. It is important to note that due to the nature of employer benefits programs, employers often choose to market their mental health services benefit at specific time points, such as open enrollment periods, which leads to an increase in sign-ups. However, not everyone needs or wants mental health care at the time they receive the email advertising the benefit. Thus, it is possible that people initiate with a mental health digital platform before they need or are ready to engage in care. Or, if they do not sign up at the time of the email, they may forget about the program at a time when they do need care. These findings underscore the need to continue adapting digital health and other digital resources in ways that engage broader populations of users curious about mental health resources and ensure that people know how to access care if they need it. Our study did identify several factors that were associated with increased use of the platform, including the use of a coach or therapist, older age, identifying as a woman, baseline symptom severity, and presenting primary concerns of finances, physical well-being, professional life, and relationships.

A key finding of our study was that the use of coaching or therapy was associated with increased self-directed digital resource use. This relationship was even stronger for individuals with elevated GAD-7 and PHQ-9 scores. Higher self-directed use in this population may be the result of provider encouragement. Coaches and therapists seeing participants with elevated GAD-7 and PHQ-9 scores might be inclined to recommend additional use of digital health resources outside of one-on-one sessions, resulting in more self-directed use for those groups. Thus, additional education for platform mental health coaches and therapists about available digital support tools and their quality and effectiveness might facilitate greater user engagement in app-based resources. Training for the network of platform providers could also include information on the barriers and facilitators that sustain engagement in digital resources. Indeed, previous research has highlighted the impact clinicians can have on digital health interest, use, and engagement [29-31]. While clinicians may be recommending self-directed strategies and driving use, alternatively, it may be that when a participant engages in 1 modality of the platform, they are more likely to become “super users” across multiple modalities. Future research is needed to disentangle the relationship between live support and self-directed resource engagement.

Furthermore, certain topics of focus were more associated with self-directed resource use, including finances, physical well-being, professional life, and relationships. Participants whose primary chosen topic was “[their] emotions” were less likely to engage with self-directed digital resources, though almost half (48.1%, n=224) of this group used one-on-one modalities. Given that higher baseline depression was also associated with less self-directed digital engagement, perhaps features of depression, such as lack of motivation, energy, or concentration, could decrease the likelihood of using these resources, even when the need for them is higher. This result is consistent with findings from a systematic review that human factors, such as severe mental health symptoms, can be a barrier to engagement [15]. This may also be a feature of the personalized care recommendations, which were more likely to suggest users work with a provider one-on-one if they were experiencing higher levels of distress. Future research may be helpful in determining how to make digital health self-directed resources more appealing for individuals struggling with low mood who are not already engaging in therapy or coaching. Research is also needed to better determine the circumstances when self-guided care is a clinically effective stand-alone approach for people with depressive symptoms.

Several sociodemographic characteristics, including identifying as a woman, higher education, and older age, were also associated with greater self-guided resource use. The extant literature has demonstrated that women are more likely to receive mental health treatment in general [32,33] and to use digital mental health resources specifically [34]. Our finding extends previous literature by revealing that at the intersection of digital health and mental health treatment, women users are more likely to engage with the technology than men. Higher education was also related to increased use of self-directed resources, as well as overall increased use of the platform more generally. Some research indicates that education is a significant predictor of mental health service use [35-37]. Finally, older age was associated with more self-directed resource use, though given the restricted age range of this sample, this result may be more challenging to interpret. It is possible that people in their 30s and 40s are more time-constrained by career and caregiving responsibilities and may be more likely to engage in mental health services they can access when it is convenient to them.

This study had several limitations with regard to the sample and restrictions of data. First, participants were eligible for the study if they had matched with a provider or used at least 1 digital mental health resource. This inclusion criteria likely selected participants who were more predisposed to engaging with digital mental health resources, possibly biasing our sample to more resource use. In addition, the digital mental health resource use that made participants eligible for inclusion in the study is included in the total number of times a participant used these resources, likely inflating the count of times used. Next, our sample may not be generalizable to other samples, given that we used participants from companies that were contracted with 1 mental health platform. Thus, the sample was limited to working adults with benefits-eligible positions, which skews towards a higher level of socioeconomic status.

Another limitation of this study was our inability to determine the sequence of events for each participant, for example, whether they engaged in a coaching session and then self-directed resource use or vice versa. This information would be helpful in further breaking down predictors of digital health use. For example, do sessions with a coach then lead to subsequent self-directed resource use, or does an attempt at self-directed resources lead to a request for additional live support by coaching or therapy? Future research can disentangle the timing of the use of various modalities. Third, the platform design and user experience may have driven engagement to some extent, and future research is needed to explore intervention-level factors (eg, ease of use, perceived effectiveness) that may affect treatment engagement.

Next, we used a definition of engagement based on the number of times a participant used the self-directed digital mental health resources. In addition to the volume of care used, it is important to understand whether users are engaging in the right level of care based on their needs. Although we found alignment between self-guided resource use and user needs (eg, users with lower depression symptoms and less mental health-focused topics of concern engaged in resources on their own, while users with higher depression and anxiety were more likely to engage with resources in combination with one-on-one care), additional data could provide a more nuanced look at engagement. For example, future studies could examine whether users engaged in their recommended care pathway and if they saw improved mental health outcomes as a result of engaging.

### Conclusion

Digital mental health platforms, including those using a stepped-care model, have the potential to deliver appropriate and evidence-based care to large populations of individuals in need. However, these platforms are affected by similar challenges related to engagement that plague many of the high-quality, evidence-based psychological treatment modalities currently available. This study revealed that users were more likely to use self-directed digital mental health resources if they were also engaged in at least 1 coaching or therapy session, which suggests that the key to successful engagement with digital therapeutics may lie in additional live support.

## Abbreviations

GAD-7: 7-item Generalized Anxiety Disorder Questionnaire
MHLS: Mental Health Literacy Scale
PAM-MH: Patient Activation Measure for Mental Health
PHQ-9: 9-item Patient Health Questionnaire
RR: rate ratio
WHO-5: 5-item World Health Organization Well-Being Index

## Data Availability

The data set analyzed in this study is not publicly available as it is proprietary information from Modern Health, but are available from the corresponding author upon reasonable request.
